# Photon-Counting Coronary CT Angiography in Asymptomatic Patients With Extreme Coronary Artery Calcium Score

**DOI:** 10.1016/j.jacadv.2025.102553

**Published:** 2026-01-21

**Authors:** Arthur Shiyovich, Camila V. Blair, Jonathan A. Aun, Avinainder Singh, Rhanderson Cardoso, Daniel Huck, Ron Blankstein

**Affiliations:** Division of Cardiovascular Medicine, Department of Medicine, Brigham and Women's Hospital, Harvard Medical School, Boston, Massachusetts, USA

**Keywords:** asymptomatic coronary artery disease, coronary artery calcium, coronary CT angiography, photon-counting computed tomography

Coronary artery calcium (CAC) testing is increasingly utilized for risk assessment in preventive cardiology. A high CAC score is associated with adverse cardiovascular outcomes, with this risk being particularly pronounced among individuals with extreme CAC levels (eg, >1,000 AUs), even in those who are asymptomatic.^1^ Despite recommendations for aggressive preventive measures and avoidance of routine testing in asymptomatic patients, data regarding this specific subgroup with extensive CAC remain scarce, and many individuals still undergo additional evaluations, including functional testing, coronary computed tomography angiography (CCTA), and even coronary angiography. CCTA in patients with a large burden of coronary calcification is often avoided as it is limited by blooming artifacts that can obscure lumen assessment and reduce diagnostic confidence. These artifacts may also affect the accuracy of fractional flow reserve derived from CT (FFR-CT).^2^^,^^3^ However, compared with conventional CT, photon-counting CT (PCCT) provides improved spatial resolution, reduces artifacts, and improves image quality, particularly in high-CAC patients, potentially aiding stenosis assessment in this high-risk group.^3^^,^^4^

**What is the clinical question being addressed?**

What is the diagnostic yield of photon-counting CT angiography in asymptomatic patients with extreme (>1,000) coronary artery calcium?

**What is the main finding?**

Photon-counting CCTA was diagnostic in all cases, demonstrated predominantly nonobstructive or functionally insignificant disease, and reliably excluded high-risk coronary anatomy, reducing the need for further testing.

Data on PCCT utility in asymptomatic patients with extreme CAC scores are limited. This study examines real-world PCCT findings in this group.

We evaluated asymptomatic patients who were referred for PCCT CCTA between February 2024 and March 2025 following the identification of extreme CAC (AU >1,000). Patients with symptoms or any history of prior coronary revascularization or events—including percutaneous coronary intervention, myocardial infarction, or coronary artery bypass grafting—were excluded. Patients were identified via the Research Patient Data Registry by cross-referencing CAC and CCTA studies. The Mass General Brigham Institutional Review Board approved the study with a waiver of informed consent.

CCTA imaging was performed using a PCCT scanner (NEOTOM Alpha, Siemens Healthineers) following institutional and Society of Cardiovascular Computed Tomography guidelines and using an ultra–high-resolution mode (0.2 mm slice thickness). Nitroglycerin and beta-blockers were administered as indicated. Scans were interpreted by level III-trained readers in accordance with the Coronary Artery Disease–Reporting and Data System (CAD-RADS) 2.0 guidelines, including detailed per-vessel and per-segment assessment. FFR-CT (performed by HeartFlow Inc) was performed at the discretion of the CCTA reader, sometimes in consultation with the treating cardiologist, and was considered negative when the value was ≥0.80. Invasive coronary angiography (ICA) was performed at the discretion of the treating cardiologist, while instantaneous wave-free ratio (iFR) measurements were obtained at the discretion of the interventional cardiologist and were considered negative when ≥0.89.

Nineteen patients were analyzed (mean age: 64.5 years, 32% women). Cardiovascular risk factors were common, with a mean of 2.8 risk factors per patient (median = 3). Dyslipidemia was universal, while hypertension (68%), diabetes (16%), and prediabetes (21%) were also prevalent. Seven patients (37%) had a family history of premature CAD, and 4 had elevated lipoprotein(a). The mean body mass index was 28.6 ± 5.5 kg/m^2^, and the mean CAC score was 1,510 AU (median 1,389, IQR: 1,107-1,919).

All CCTA scans were of diagnostic quality, meaning that all segments were deemed evaluable for stenosis by the interpreting reader at the time of image assessment and independently confirmed by a second reader during retrospective review. Overall, 74% of patients were found not to have severe coronary stenosis. The distribution of CAD-RADS scores was as follows: CAD-RADS 2 in 6 patients (32%), CAD-RADS 3 in 8 patients (42%), CAD-RADS 4 in 4 patients (21%), and CAD-RADS 5 in 1 patient (5%). Among those with CAD-RADS 3 and 4, 7 patients underwent FFR-CT (5 of 8 with CAD-RADS 3 and 2 of 4 with CAD-RADS 4), all of which were negative for functionally significant stenosis, with values ranging from 0.80 to 0.93, all with translesional drops ≤0.10 (Figure 1).

**Figure 1:**
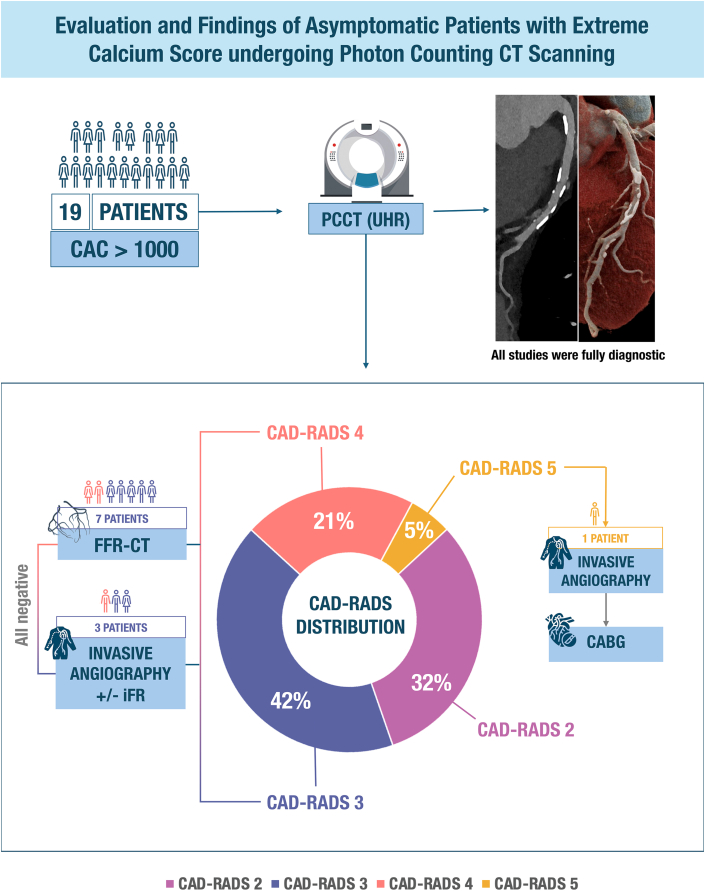
**Evaluation of Asymptomatic Patients With Extreme Coronary Calcium Undergoing Photon-Counting Computed Tomography** Nineteen asymptomatic patients with CAC scores >1,000 underwent ultra–high-resolution PCCT. All PCCT examinations were fully diagnostic. The distribution of coronary artery disease severity as assessed by CAD-RADS was CAD-RADS 2 in 32% of patients, CAD-RADS 3 in 42%, CAD-RADS 4 in 21%, and CAD-RADS 5 in 5%. Seven patients underwent FFR-CT, all demonstrating no physiologically significant stenosis. Three patients underwent invasive coronary angiography with or without iFR, all of which were negative for flow-limiting disease. One patient with CAD-RADS 5 proceeded to invasive angiography confirming severe multivessel coronary artery disease and subsequently underwent CABG. CABG = coronary artery bypass grafting; CAC = coronary artery calcium; CAD-RADS = Coronary Artery Disease–Reporting and Data System; FFR-CT = fractional flow reserve derived from computed tomography; iFR = instantaneous wave-free ratio; PCCT = photon-counting computed tomography; UHR = ultra–high-resolution.

Four patients underwent ICA: one CAD-RADS 5 patient, whose ICA findings were consistent with CCTA and led to coronary artery bypass grafting; 2 CAD-RADS 3 with negative FFR-CT, found to have nonobstructive CAD (one had iFR); and one CAD-RADS 4 patient who was found to have moderate stenosis and negative iFR (0.92). This patient did not undergo FFR-CT. None of these patients underwent percutaneous coronary intervention.

This study assessed the role of PCCT-based CCTA in asymptomatic patients with extreme CAC scores in a real-world clinical setting. The diagnostic yield was high, with most exhibiting nonobstructive or hemodynamically insignificant disease, highlighting PCCT’s capability to provide a reliable assessment despite significant CAC. Overall, these findings are consistent with previous studies demonstrating enhanced PCCT performance in patients with high CAC.^3^, ^4^, ^5^

The limitation of this study includes a small sample size and retrospective design, both of which may limit external validity. Nevertheless, our findings show that PCCT can provide diagnostic quality images and that most patients did not require further testing post-CCTA.

In summary, CCTA using PCCT, supplemented by FFR-CT when indicated, can reliably exclude high-risk coronary anatomy and reduce the need for further diagnostic testing. Additional studies with larger sample sizes are warranted to assess the impact of PCCT on clinical outcomes in this population with extreme CAC.

## Funding support and author disclosures

Dr Shiyovich has received honoraria from Pfizer and serves as a consultant for Artrya Inc. Dr Singh is supported by 10.13039/100000002NIH Grant (5T32HL007604-39). Dr Huck is supported by 10.13039/100000968AHA Career Development Award (23CDA1037589). Dr Blankstein has received research support/consultant from Amgen Inc, Novartis Inc, Heartflow Inc, and Nanox AI and consultant for Siemens Inc, and Caristo Inc. Dr Cardoso has received research support from Cleerly. All other authors have reported that they have no relationships relevant to the contents of this paper to disclose.
